# Estimating Glomerular Filtration Rate in Older People

**DOI:** 10.1155/2014/916542

**Published:** 2014-03-20

**Authors:** Sabrina Garasto, Sergio Fusco, Francesco Corica, Maria Rosignuolo, Antonio Marino, Alberto Montesanto, Francesco De Rango, Marcello Maggio, Vincenzo Mari, Andrea Corsonello, Fabrizia Lattanzio

**Affiliations:** ^1^Unit of Geriatric Pharmacoepidemiology, Italian National Research Center on Aging (INRCA), C. da Muoio Piccolo, 87100 Cosenza, Italy; ^2^Department of Clinical and Experimental Medicine, University of Messina, 98122 Messina, Italy; ^3^Department of Cell Biology, University of Calabria, 87036 Rende, Italy; ^4^Department of Geriatric Rehabilitation, University-Hospital of Parma and Section of Geriatrics, Department of Clinical and Experimental Medicine, University of Parma, 43100 Parma, Italy; ^5^Unit of Clinical Pathology, Italian National Research Center on Aging (INRCA), 87100 Cosenza, Italy; ^6^Scientific Direction, Italian National Research Center on Aging (INRCA), 60127 Ancona, Italy

## Abstract

We aimed at reviewing age-related changes in kidney structure and function, methods for estimating kidney function, and impact of reduced kidney function on geriatric outcomes, as well as the reliability and applicability of equations for estimating glomerular filtration rate (eGFR) in older patients. CKD is associated with different comorbidities and adverse outcomes such as disability and premature death in older populations. Creatinine clearance and other methods for estimating kidney function are not easy to apply in older subjects. Thus, an accurate and reliable method for calculating eGFR would be highly desirable for early detection and management of CKD in this vulnerable population. Equations based on serum creatinine, age, race, and gender have been widely used. However, these equations have their own limitations, and no equation seems better than the other ones in older people. New equations specifically developed for use in older populations, especially those based on serum cystatin C, hold promises. However, further studies are needed to definitely accept them as the reference method to estimate kidney function in older patients in the clinical setting.

## 1. Introduction

Chronic kidney disease (CKD) is an important epidemic and public health problem, resulting in end-stage renal disease (ESRD) and increased risk of morbidity and mortality [1]. Hence, early identification and management of CKD patients may delay the progression of renal disease. CKD is currently classified into five stages based on glomerular filtration rate (GFR) as recommended by many professional guidelines (Table 1) [2]. CKD is defined as a GFR below 60 mL/min/1.73 m^2^ or by the presence of kidney damage for 3 or more months. Conversely, individuals with a GFR from 60 to 89 mL/min/1.73 m^2^ without kidney damage are classified as “decreased GFR.” In UK, the prevalence of CKD stages 3–5 (GFR < 60 mL/min/1.73 m^2^) is estimated to be 8.5%, and based on a review of 26 studies, a prevalence of CKD of 7.2% in patients aged >30 years and of 8% in patients aged >64 years was reported [3]. The most important Italian studies about CKD prevalence are the GUBBIO and INCIPE studies. The first one included 4,574 subjects aged 18–95 years from Umbria district (Central Italy) and showed a prevalence of CKD stages 3–5 of about 5.7% in men and 6.2% in women [4]. The INCIPE study [5] included subjects aged ≥ 40 years and showed a prevalence of CKD (stages 1–4) that is equal to 12.7%. After adjusting the analysis for age and gender, the prevalence of stage 3 CKD was lower in Italy (13.2%) compared to the US population enrolled in the National Health and Nutrition Examination Survey (NHANES) (20.3%).

In USA, the prevalence of CKD based on data from the third NHANES (NHANES III) was 11% (3.3% with stage 1; 3.0% with stage 2; 4.3% with stage 3; 0.2% with stage 4; and 0.2% with stage 5). In this study, a graded increase in the prevalence of CKD was shown at older age groups [6]. In a related analysis using data from NHANES III, the highest prevalence (45%) was found among subjects aged 80 years or more [7]. The lower prevalence in Italy was related to a lower rate of the main risk factors for kidney disease, such as diabetes, obesity, and metabolic syndrome.

The association between age and incident CKD was investigated among community-dwelling participants who were part of the Framingham Offspring Study (mean age at baseline: 43 years). For every 10-year increase in age, the odds ratio for developing incident CKD was 2.56 [8]. CKD is associated with adverse outcomes such as disability [9], cachexia [10], cardiovascular disease (CVD) [11], diabetes mellitus [12], hospitalization, and death [11]. Accurate estimation of GFR is important for detecting and staging CKD, determining drug dosages, and stratifying risk. Creatinine clearance and other reference procedures, such as clearance of inulin, ^125^I-iothlamate, ^51^Cr-EDTA, or iohexol, are used to determine GFR. In the oldest subjects, the above mentioned techniques are not easily applicable and a 24-hour urine collection for creatinine clearance is often impracticable [13]. For these reasons, some equations based on serum creatinine, age, race, and sex are used to obtain an estimation of GFR (eGFR). Therefore, the aim of this review was to summarize age-related changes in kidney structure and function, methods for estimating kidney function, and the impact of reduced kidney function on geriatric outcomes, as well as the reliability and applicability of eGFR in older patients.

## 2. Age-Related Changes in Kidney Structure and Function

**Figure 1:**
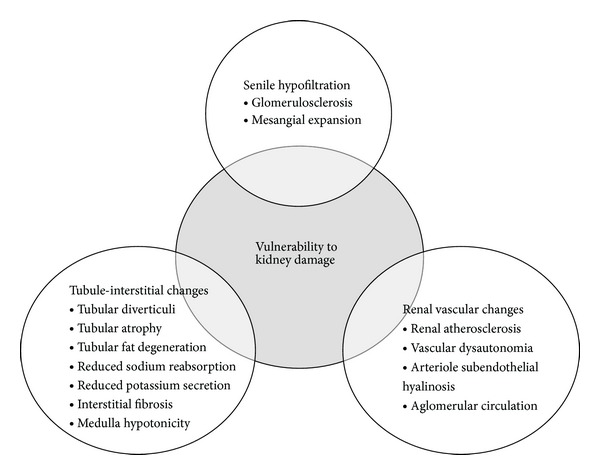
Summary of age-related changes in kidney structure and function. Data reported from Musso & Oreopoulos, Nephron Physiology 2011.

The aging process determines important modifications of kidney structure and function, such as kidney vasculature, filtration, and tubule-interstitial function (Figure 1). Overall, aging is associated with a loss of renal mass by about 20–25% from 30 to 80 years of age [14], and the length of the kidney decreases by 15% from 17 to 85 years of age [15]. At the microscopic level, the aging human kidney is characterized by increased fibrosis, tubular atrophy, and arteriosclerosis [16, 17]. In the autopsy study by Neugarten et al. [18], older age was associated with increased numbers of sclerotic glomeruli and interstitial fibrosis, with a loss of about 20 to 30% of the glomeruli present in younger adults. The aging kidney exhibits an increase of mesangium, as well as the obliteration of some juxtamedullary nephrons followed by the formation of a direct channel between afferent and efferent arterioles (i.e., aglomerular circulation). Small arteries and arterioles present intimal thickening and atrophy of the media which contribute to dysfunction of the autonomic vascular reflex [19]. The most relevant age-related tubule-interstitial changes are the formation of tubular diverticuli, atrophy, fat degeneration, interstitial fibrosis, and medullary hypotonicity [19].

Such morphological changes determine important functional alterations and make the aging kidney more vulnerable to the development of kidney disease (Figure 1). Indeed, it has shown an age-related reduction in the clearance of inulin (13%–46%) [16, 20–23]. In the Baltimore Longitudinal Study of Aging (BLSA), a decrease in creatinine clearance of 0.75 mL/min/year was observed in subjects aged 30 to 90 years, although one-third of them had no decrease in renal function for up to 25 years. Since older hypertensive patients were not excluded from the BLSA sample, it was not possible to disentangle the effect of aging on kidney function from that of hypertension [24]. Decreasing filtration during aging is accompanied by a decrease in creatinine production due to the age-related loss of muscle mass (sarcopenia), and consequently serum creatinine does not necessarily increase with the progressive decrease in filtration [25]. For this reason, GFR measurement is generally adjusted for body surface area.

Aging kidney progressively loses its ability to maintain sodium/potassium balance due to the reduced tubular sodium secretion and potassium absorption [19]. Sodium output and fractional excretion of sodium are increased in old subjects, due to a diminished response of the ascending loop of Henle to renin and aldosterone. However, reduced filtration and tubular secretion significantly slow the elimination of a salt load in older people. Additionally, medullary hypotonicity significantly contributes to reduced ability of aging kidney to concentrate the urine. Finally, both potassium secretion and urea reabsorption are reduced during aging [19].

The kidney also plays an important role in synthesis, metabolism, and elimination of different hormones. While CKD naturally progresses with hormonal disorders [26], the aging process* per se* seems to play a minor role in endocrine kidney functions.

Kidneys are the primary sources of erythropoietin (EPO). Peritubular fibroblasts in the renal cortex are the main site of EPO synthesis, which is controlled by hypoxia-inducible transcription factors (HIFs) [27]. EPO is an essential factor for the viability and proliferation of erythrocytic progenitors. Proximal tubular function is preserved in healthy older people and their serum EPO levels are usually normal [19] or slightly increased perhaps as a compensatory response to age-related subclinical blood loss, increased erythrocyte turnover, or increased EPO resistance. However, EPO levels are unexpectedly lower in anemic older patients compared to younger ones, suggesting a blunted response to low hemoglobin [28].

Vitamin D is necessary to maintain calcium homeostasis and optimal bone health [29]. The main circulating form of vitamin D is 25-hydroxyvitamin D (25[OH]D) (calcidiol), which requires activation by renal 1*α*-hydroxylase to form the metabolically active form of vitamin D, 1,25-dihydroxvitamin D (1,25[OH]_2_D) (calcitriol). Parathyroid hormone (PTH) increases activity of the renal 1*α*-hydroxylase in response to low calcium levels. Age* per se* does not affect PTH and active vitamin D levels, which are usually normal in healthy older people [19], whereas CKD results in decreased kidney mass and compensatory elevation in PTH [30].

A progressive reduction in renal function is linked to alterations in thyroid hormone levels and/or metabolism, resulting in high prevalence of subclinical hypothyroidism and the low T3 syndrome [31]. This syndrome is mainly characterized by a decrease in total (T3) and free triiodothyronine (fT3) plasma concentration, whilst thyroid-stimulating hormone (TSH) and T4 remain in the normal range. Recent studies suggest that as many as 80% of patients with ESRD present low T3 levels and as many as 20 to 25% are subclinically hypothyroid [32].

Kidney is the major site of insulin clearance from the systemic circulation, removing approximately 50% of insulin in the peripheral circulation. Insulin clearance by the kidney is accomplished by glomerular filtration and proximal tubular uptake and degradation [33]. The age-related decline in kidney function leads to reduced insulin clearance, which is partly offset by diminished glucose tolerance due to defective insulin secretion and action during aging.

Finally, alterations of sex steroid production and metabolism (leading to primary hypogonadism and disturbances of the hypothalamic-pituitary axis) are observed when moderate GFR reductions arise. As many as 40 to 60% of CKD stage-5 men have been reported to be hypogonadal on the basis of low concentrations of total and free testosterone [34].

## 3. Methods for Estimating Kidney Function

Measurement of renal function is important in the diagnosis and management of renal diseases. GFR is the standard measure of renal function. GFR is the rate at which substances are filtered from the blood of the glomeruli into Bowman's capsules of the nephrons. Any substance freely filtered by the glomerulus and not subsequently secreted, reabsorbed, or metabolized by the distal parts of the renal system has a clearance equivalent to the GFR. It correlates with renal damage in the kidneys of patients with chronic kidney disease, and it therefore reflects overall renal functional capacity. In addition, most functions of the kidney, including endocrine ones (i.e., 1,25-dihydroxyvitamin D and erythropoietin synthesis), are directly related to GFR. In addition, appropriate dosing of drugs excreted by the kidney depends on accurate estimation of GFR. For these reasons, GFR is the most widely accepted measurement for assessing the overall function of the kidney [35].

Conventional techniques for estimating GFR use the principle of renal clearance of various markers of GFR, including creatinine, cystatin C, inulin, and radiocontrast agents (e.g., iothalamate and iohexol). Renal clearance techniques involve measuring blood and urine concentrations of either endogenous (e.g., creatinine, cystatin C) or exogenous (e.g., inulin, radiocontrast agents) substances and calculating GFR from the ratio of urine to plasma concentrations of the marker, multiplied by the urine flow rate. These methods however are not always easily suitable in clinical practice. For this reason, equations for estimating GFR (estimated GFR, eGFR) were developed. In this section, we will discuss feasibility and reliability of these methods, with special attention to eGFR equations in older people.

### 3.1. Clearance of Exogenous Markers

#### 3.1.1. Inulin

Inulin is an inert polyfructose sugar that does not bind to plasma protein, is freely filtered by the kidney, does not undergo metabolism, tubular secretion, or absorption, and is therefore rapidly excreted into the urine by glomerular filtration only. Measuring inulin clearance needs an indwelling intravenous cannula, and urinary catheter must be in place. The technique involves oral water loading (15–20 mL/kg) and intravenous loading dose of inulin of 30 to 50 mg/kg followed by a continuous infusion until establishing a steady-state plasma concentration of 15 to 20 mg/dL. The bladder is usually flushed with air to eliminate any pooled urine. After a 1 h equilibration period, three to four 30-min urine collections with midpoint (or flanking) blood specimens are obtained for measurement of blood and urine concentration of inulin [35]. Clearance is computed for each urine collection period, and the results are averaged [36]. It is generally accepted that inulin clearance provides the most accurate available determination of GFR [37]. However, it is rarely used in clinical setting because it is cumbersome for several reasons: inulin is expensive, its commercial sources are limited, it must be dissolved by boiling before use, and the laboratory assay is complex and expensive [35]. Moreover, it is extremely uncomfortable for older patients and increases the risk of urinary tract infections from urinary catheter. When spontaneous voiding is used, incontinence or retention may increase the risk of error due to incomplete urine collections [38].

#### 3.1.2. Iothalamate

Iothalamate is commonly administered as a radioactive iodine label for ease of assay after small doses; it is most commonly administered using bolus subcutaneous injection [38]. ^125^I-Iothalamate has been widely adopted for measurement of GFR. To block thyroidal uptake, cold iodine is administered at the time of ^125^I-iothalamate administration, thus precluding its use in people with known allergies to iodine [38].

#### 3.1.3. Iohexol

In order to avoid the use of radioactive compounds, techniques have been developed aimed at detecting low levels of iodine compounds in the urine. This has allowed the use of nonradiolabeled iodinated contrast agents, such as iohexol. The assay of these agents can be obtained by high performance liquid chromatography (HPLC) [39], which is unfortunately a very expensive and time consuming method [40–43]. For these reasons, a new technique has been proposed based on X-ray fluorescence. However, such method is less sensitive than HPLC, necessitating the administration of significantly larger doses of iohexol, thus leading to increased risk of nephrotoxicity and adverse reactions [38, 40, 44, 45]. As the iothalamate, iohexol cannot be used in patients with allergy to iodine.

#### 3.1.4. Other Exogenous Markers

Among other markers, ^51^Cr-EDTA consistently underestimates inulin clearance, probably because of tubular reabsorption [46]. Diethethylenetriaminopentaacetic acid (DTPA), an analog of EDTA, usually labeled with ^99m^Tc, may undergo extrarenal elimination and can bind to plasma proteins to a nonpredictable extent, leading to imprecision and bias [38].

### 3.2. Clearance of Endogenous Markers

Measurement of the clearance of endogenous filtration markers, such as creatinine, is widely used in clinical setting.

#### 3.2.1. Creatinine and Creatinine Clearance

Creatinine is a metabolic product of creatine and phosphocreatine arising from the muscle compartment. Thus creatinine is directly related to muscle mass and undergoes little daily change [47]. However, its production may change over time if considering longer periods in which major changes occur in body composition [48]. Creatinine has a low molecular weight (113 D), does not bind to plasma proteins, and is freely filtered by the glomerulus. There is also a little, but not negligible, quote of secretion of creatinine by the renal tubule. The proportion of creatinine excreted by tubular secretion increases with the reduction of renal function [49], and this has important clinical implications because the GFR may decrease more rapidly than creatinine clearance, which may therefore overestimate kidney function. A small extrarenal elimination has been demonstrated likely linked to the degradation of creatinine by intestinal bacteria and therefore influenced by antibiotics [50].

Current recommendation for creatinine measurement suggests the use of a standardized method based on modified Jaffé reaction, able to separate creatinine from noncreatinine chromogens [51, 52]. Unfortunately, creatinine alone is not very sensitive. Indeed, a 50% reduction in GFR is necessary so that the values of creatinine begin to rise [53]. Additionally, circulating creatinine may be falsely low in patients with reduced muscle mass: older patients often have decreased renal function with normal circulating levels of creatinine, which has been referred to as concealed renal failure [54].

To obtain creatinine clearance, a long urinary collection period—6 to 24 h—is used to avoid the requirement for water loading and, in the steady state, a single blood sample obtained either at the beginning or the end of the collection period may be assumed to represent the average serum concentration during the urine collection. Timed collections are subject to errors in older patients, due to inaccurate record of time and incomplete urine collection in patients with incontinence [38]. The tubular secretion of creatinine is extremely variable and does not allow the use of a constant correction factor [55]. Variability in creatinine clearance measurement also depends on age, gender, and muscle mass [56]. Even the dietary intake is a source of variability: creatine derived from ingested meat is converted into creatinine and may result in increases up to 30 per cent of its total excretion [57].

#### 3.2.2. Cystatin C

Cystatin C (CysC) is a single chain basic protein with low molecular weight (13 kD) produced by all nucleated human cells, whose circulating concentrations can be easily determined by an automated particle-enhanced immunoturbidimetric method [58]. CysC is mainly filtrated by the kidney [59, 60], and renal clearance of CysC is 94% of the renal clearance calculated using the Cr51EDTA clearance [61, 62]. However, CysC also undergoes tubular catabolism and reabsorption. Other factors affecting the production of CysC include the use of systemic glucocorticoids [63] and thyroid dysfunction [64, 65]. CysC was proposed as a marker of GFR potentially superior to serum creatinine [60]. A meta-analysis of 46 cross-sectional studies including adults and children suggested the superiority of CysC compared to serum creatinine and to creatinine-based equations in the estimation of GFR [60]. CysC and microalbuminuria are considered early markers of kidney damage [66]. Finally, CysC was the best predictor of kidney failure and death from cardiovascular disease in a longitudinal cohort study of 4637 older people [67]. Nevertheless, even CysC could be affected by changes in body composition. Indeed, fat-free mass, a parameter inversely related to age, affects CysC level, and in older patients with chronic kidney disease CysC-based GFR estimation improves when fat-free mass is taken into account [68].

### 3.3. GFR Equations

Equations have been proposed in order to provide the physician an easy way of calculation and an accurate estimation of kidney function (Table 2). The Cockcroft and Gault equation [69] was the first published and still widely used. However, it does not take into account the variability of creatinine production [69, 70], and it systematically overestimates the GFR in obese or edematous patients [70].

The Modification of Diet in Renal Disease Study (MDRD) equations were derived on the basis of measured ^125^I-iothalamate clearance and were normalized to 1.73 m^2^ body surface area (BSA) [71]. Their use has been endorsed by national professional health care organizations [52]. Additionally, the original MDRD equations were reexpressed to account for the difference resulting from the standardization of serum creatinine measurements to the isotope dilution mass spectrometry (IDMS) reference method [72]. Nevertheless, MDRD equations were found to lose accuracy in selected subgroups of patients, such as those with normal renal function, type 1 diabetes, elderly, and kidney transplant recipients (i.e., subgroups not included in the MDRD study population) [73–75]. Indeed the MDRD Study equations were developed in people with CKD, and as such their major limitations are imprecision and systematic underestimation of measured GFR at higher levels of kidney function [76].

The Chronic Kidney Disease Epidemiology Collaboration (CKD-EPI) equations were developed in an attempt to improve the estimation of GFR in patients older than 70 years [77]. Creatinine-based CKD-EPI equation is based on standardized serum creatinine [77, 78]. It was found to be as accurate as the MDRD equation at GFR less than 60 mL/min/1.73 m^2^ and more accurate than the MDRD equation at higher GFR values [77, 79]. To overcome the bias due to variability of serum creatinine, equations based on CysC alone (CKD-EPI_CYS_) or in combination with creatinine (CKD-EPI_CR-CYS_) have been developed. Overall, the CKD-EPI_CR-CYS_ equation had better precision and accuracy than that based on creatinine alone or CysC alone [80]. Nevertheless, even CKD-EPI study population included relatively few participants older than 70 years of age.

Recently, two eGFR equations were developed and validated in a population of older adults aged 70 years or more enrolled in the Berlin Initiative Study (BIS): the BIS1 equation based on serum creatinine alone and the BIS2 equation based on both serum creatinine and cystatin C [81]. BIS equations showed excellent agreement with directly measured GFR [81]. A study comparing the performances of BIS-1, MDRD, and CKD-EPI equations in estimating GFR in older patients showed that BIS-1 was the most reliable for assessing renal function in older white patients, especially in those with CKD stages 1 to 3 [82]. In old people (mean age: 85 years), CKD-EPI_CR-CYS_ and BIS2 equations showed better accuracy compared to MDRD, CKD-EPI, and BIS1 estimates.

## 4. Estimated GFR and Outcomes Relevant to Older Patients

Although the overall prevalence of CKD is roughly 10% in the general population, it increases with age [2]. Additionally, CKD is associated with several comorbid conditions in older people, such as cardiovascular disease and disability, which in turn increase the risk of hospitalization and death [3, 4].

Several studies have demonstrated that a reduction of eGFR is associated with lower scores in subjective physical function and physical activity scales [83]. In an elderly population study, declining eGFR was associated with increasing risk of worsening disability, defined as the loss of ≥ 1 Activities of Daily Living (ADL) over the 6-year follow-up, at GFR below 60 mL/min [84]. In the Cardiovascular Health Study cohort, including 5,888 persons aged 65 years or older [85], the cross-sectional prevalence of a limitation in ADL was 12% among participants with CKD compared to 7% among participants without CKD. Finally, frailty, a condition characterized by a decline in physical function and functional capacity predisposing to disability, is highly prevalent among patients with CKD [86].

Osteoporosis and CKD are both common conditions in older adults and may be associated with substantial morbidity [87]. In the NHANES III study, a double risk of hip fractures was observed among persons with eGFR below 60 mL/min/1.73 m^2^ compared to the general population [88]. Additionally, several studies have shown that hypocalcemia, hyperphosphatemia, hyperparathyroidism, vitamin D deficiency (both 25-OH and 1,25-OH vitamin D), and metabolic acidosis play a key role in increasing the risk of fractures in older people [89]. Disorders of mineral-bone metabolism leading to abnormal bone architecture and fracture may in part explain the relationship between CKD and low physical function.

Cognitive impairment has been frequently observed in patients with CKD especially in older subjects [90]. CKD is related to a wide range of deficits in cognitive functioning, including verbal and visual memory and organization, and components of executive functioning and fluid intellect. Small vessel disease, which is known to contribute to the pathophysiology of CKD [91], can lead to cerebral ischemic lesions, both in the form of silent or subclinical cerebral infarcts or white matter lesions, increasing the risk of cognitive decline and dementia [92]. In the Cardiovascular Health Study, a 37% higher risk of incident dementia was found among older adults with CKD during a median follow-up period of 6 years [93]. Patients in all stages of CKD have a higher risk for development of cognitive impairment and this may be a major determinant in their quality of life and cognitive impairment is associated with an increased risk of death in dialysis patients [94].

Stroke is the most frequent neurological disease and represents a continuously evolving medical and social problem [95]. A Taiwanese study showed that CKD itself may represent a causal risk factor for stroke beyond traditional cardiovascular risk factors. Indeed, patients primarily affected by CKD have higher risk of stroke compared to the general population [96]. Thus, patients with chronic renal failure should be carefully monitored for prevention of stroke regardless of the presence and severity of traditional cardiovascular risk factors. MRIs studies have demonstrated that patients with CKD have a higher prevalence of subclinical brain infarcts and deep white matter lesions, even after adjusting to traditional risk factors such as smoking, hypertension, and diabetes [97]. There are only few data on the impact of renal dysfunction on mortality in patients suffering from stroke. In the study of Hojs Fabjan et al. [65], decreased eGFR was associated with higher short-term (in-hospital) mortality among patients with ischemic stroke.

Depression is frequently observed in elderly patients with CKD [98]. This is of great importance because the presence of depression in CKD is associated with poorer outcomes such as hospitalizations, progression to dialysis, and increased morbidity and mortality [99]. About 20% to 30% of patients with CKD have clinical depression [98]. In a retrospective study of elderly patients with non-dialysis-dependent CKD stages 1–5 followed up for 4.7 years about 30% had depression, which was associated with significantly higher age-adjusted mortality rates [100].

Anemia is commonly related to CKD, and it is typically normocytic, normochromic, and hypoproliferative [101]. In prospective randomized controlled trials, anemia is related to mortality, nonfatal cardiovascular events, left ventricular hypertrophy, hospitalizations, and progression of kidney disease [102]. Data from Longitudinal Aging Study Amsterdam with a follow-up of 3 years indicate that anemia in older adults doubles the risk of recurrent falls [103]. This comorbidity can be particularly dangerous in older adults, since it may also lead to other negative outcomes such as impaired physical function and cognitive decline [104].

Obstructive sleep apnea (OSA) is an important and common comorbidity in patients with CKD [105]. OSA increases the risk of systemic hypertension and vascular disease [106], both of which are common complications of CKD. OSA may also accelerate the deterioration of renal function in patients with CKD directly through the effect of hypoxia on the kidney [107] or indirectly by increasing systemic blood pressure, inflammatory cytokines, and sympathetic nervous system activity [108]. This condition leads to an impairment of sleep quality and daytime function [109].

Chronic obstructive pulmonary disease (COPD) dramatically increases with age and it is a progressive, debilitating respiratory condition [110]. COPD is characterized by typical symptoms including lung airflow limitations, cough, and difficulty breathing. This condition may lead to emphysema and chronic bronchitis and plays a pivotal role in conditioning the health status and having major prognostic implication. An important study of Antonelli Incalzi et al. showed that COPD is significantly associated with CKD [111].

Cachexia is an important cause of death in elderly CKD patients, even if it is not completely clear whether malnutrition is part of a cause-effect relationship. In adult CKD patients, decreased appetite plays a major role in wasting. Wasting has also been linked to high levels of leptin and proinflammatory cytokines [112]. Malnutrition is considered to be a uremic risk factor for cardiovascular disease, leading to an increased cardiovascular mortality. Moreover, atherosclerosis is considered an inflammatory disease, and chronic inflammation may reduce the patient's appetite and increase the rate of protein depletion, wasting, and hypercatabolism [113].

When renal function declines, many drugs or their active metabolites that depend on renal excretion may accumulate with an increase of potential toxicity, and patients with renal disease may be more vulnerable to a given drug effect [114]. This necessitates dosage adjustment in order to prevent adverse drug reactions (ADRs) [115]. This is especially important in older people, who are more vulnerable to adverse drug reactions due to an increased prevalence of renal impairment (partly due to structural and functional changes in the kidney as a result of aging), polypharmacy, and frailty [116]. It is worth noting that reduced eGFR is associated with increased risk of adverse drug reactions from water-soluble drugs even when serum creatinine levels are within the normal range [25]. High risk combination of drugs in people treated with complex polypharmacy regimens deserves to be mentioned. An example of high risk combination is the simultaneous use of diuretics, nonsteroidal anti-inflammatory drugs (NSAIDs), ACE inhibitors (ACEI), and/or angiotensin receptor blockers (ARBs) (the so-called triple whammy) that may impair kidney function [117]. Older patients are at greater risk of experiencing this triple whammy effect, and although medications for hypertension and heart failure have the important potential to reduce the likelihood of stroke and myocardial infarction, much care must be taken to ensure that this is not achieved at the price of inducing renal failure, especially in the elderly [118].

## 5. Potential Issues in Estimating GFR in Older Patients

Despite the huge amount of findings described above, eGFR has some important limitations when applied to older and frail patients. Indeed, whatever is the equation used to estimate GFR, the interpretation of results obtained in older people may not be so easy.

An example of how it can be difficult to understand the meaning of these measures in frail and older patients comes from studies showing the existence of a U-shaped relationship between eGFR and mortality. By using MDRD equation, Cox et al. showed for the first time that all-cause mortality risk increased in subjects with eGFR higher than 89 mL/min/1.73 m^2^ enrolled in the Hull and East Yorkshire renal and diabetes registers [119]. An increased risk of cardiovascular events and a near significant increase in total and cardiovascular mortality were observed in octogenarians with CKD-EPI-based eGFR values of 75 mL/min/1.73 m^2^ or more enrolled in the HYVET trial [120]. All-cause mortality, but not mortality from myocardial infarction or stroke, was significantly increased in patients with MDRD-based eGFR greater than or equal to 90 mL/min/1.73 m^2^ in the large population study of the Alberta Kidney Disease Network repository [121]. Finally, a similar U-shaped relationship between eGFR and mortality was observed in the Cardiovascular Health Study with creatinine-based CKD-EPI, but not with cystatin-C-based CKD-EPI equation [122]. There are at least three potential mechanisms which could be invoked to explain this apparent discrepancy in the relationship between eGFR and mortality [123]: (i) a direct harmful effect exerted by high eGFR on kidney hemodynamics, as it has been demonstrated in obese and diabetic individuals [124, 125] (however, with this phenomenon one would expect higher eGFR to associate more strongly with progression of kidney disease than with mortality, which has not been demonstrated [123]); (ii) an effect due to unmeasured or residual confounding from other detrimental pathophysiological processes, such as excess body weight as observed in obesity [123]; (iii) high eGFR which may not only be a reflection of GFR, but also reflects inflammation, frailty, and/or muscle loss which may contribute to reducing serum creatinine. Though not definitely demonstrated, this latter mechanism is more likely to explain the observed U-shaped relationship between eGFR and mortality. Indeed, both low serum creatinine and low 24 h urine creatinine are associated with adverse outcomes [126]. Interestingly, such a U-shaped relationship was not observed with CysC-based eGFR [122]. Thus, despite the findings showing that even CysC could be affected by changes in body composition [68], CysC-based eGFR likely represents the most meaningful and reliable method to estimate kidney function in older patients.

Another potential issue is represented by the difference among kidney function estimates obtained with different equations. Structural differences between equations likely account for discrepancy. Indeed, the Cockcroft-Gault equation intends to measure the creatinine clearance, whereas all other equations listed in Table 2 are proxies of the GFR. Creatinine clearance is influenced by tubular secretion and extrarenal clearance of creatinine as well as by drugs affecting the renal handling of creatinine [127]. For this reason, creatinine clearance usually exceeds GFR, whereas Cockcroft-Gault equation usually provides lower values than GFR equations, and age and weight are main sources of discrepancy [128]. This makes Cockcroft-Gault and GFR equations not interchangeable in the estimation of renal function. Implications of the above findings are straightforward; dosing requirement of a given drug cleared by the kidney will dramatically change depending upon the equation used to obtain an estimate of kidney function. This is especially true for the Cockcroft-Gault versus MDRD- or CKD-EPI-based values and might result in underdosing and, then, lack of efficacy or overdosing and, then, risk of ADRs [129]. As a general rule, it seems reasonable to suggest adjusting kidney cleared drugs dosing according to the recommendation provided by the manufacturer and if no equation is recommended, refer to the one proved more reliable in the reference population [129]. Further studies using newly available equations specifically developed in older patients (BIS equations) [81] are needed to verify whether the greater accuracy in estimating GFR could be translated into recommendations for drug dosing.

## 6. Conclusions

The prevalence of CKD increases with age, and CKD is often associated with several comorbid conditions and adverse outcomes in older patients. Thus, the availability of an accurate method for estimating kidney function in this highly vulnerable population would be of paramount importance.

Methods based on clearance of radiolabelled or nonradiolabelled exogenous markers are expensive and not easy to apply in clinical settings. Kidney function estimate based on simple determination of serum creatinine level is hardly reliable in older patients because of the frequent loss of muscle mass secondary to age itself and aging-related conditions. Additionally, the clearance of creatinine is often biased in older patients due to inaccurate or incomplete urine collection. GFR equations may facilitate the estimation of kidney function in older patients. However, all of them have their own limitations, and no equation proved to be better than the other ones. New equations specifically developed for use in older people, especially those based on serum cystatin C, hold promises. However, few studies have been carried out to definitely accept them as the reference method to estimate kidney function in older patients in clinical practice. Further research is needed to verify whether these new equations can overcome the above described issues in prognostic stratification and dosing of kidney cleared drugs.

## Figures and Tables

**Table 1:** The kidney disease outcomes quality initiative (KDOQI) stages of kidney disease.

Stage	GFR*	Description
1	90+	Normal kidney function
2	60–89	Mildly reduced kidney function
3A 3B	45–59 30–44	Moderately reduced kidney function
4	15–29	Severely reduced kidney function
5	<15 or on dialysis	Very severe or endstage kidney failure

*All GFR values are normalized to an average body surface area (BSA) of 1.73 m^2^.

**Table 2:** Equations for estimating renal function.

Cockcroft and Gault [69]	eCCr = (140−age) × weight in kg/(72 × Scr), ×0.85 in females

Six-variable MDRD [71]	170 ∗ [Scr]^−0.999^ ∗ [age]^−0.176^ ∗ [BUN]^−0.170^ ∗ [serum albumin]^0.318^, ∗0.762 in females, ∗1.180 if black

Four-variable MDRD [71]	[186.3 × (Scr)^−1.154^ × (age)^−0.203^], ×0.742 in females, ×1.212 if black

CKD-EPI (creatinine) [77]	Female (Scr ≤ 0.7), eGFR = 144 × (Scr/0.7)^−0.329^ × (0.993)^Age^ (Scr > 0.7), eGFR = 144 × (Scr/0.7)^−1.209^ × (0.993)^Age^ Male (Scr ≤ 0.9), eGFR = 141 × (Scr/0.9)^−0.411^ × (0.993)^Age^ (Scr > 0.9), eGFR = 141 × (Scr/0.9)^−1.209^ × (0.993)^Age^

CKD-EPI (cystatin C) [80]	(Scys ≤ 0.8), eGFR = 133 × (Scys/0.8)^−0.499^ × 0.996^Age^ [×0.932 if female] (Scys > 0.8), eGFR = 133 × (Scys/0.8)^−1.328^ × 0.996^Age^ [×0.932 if female]

CKD-EPI (cystatin C-creatinine) [80]	Female (Scr ≤ 0.7) (Scys ≤ 0.8), eGFR = 130 × (Scr/0.7)^−0.248^ × (Scys/0.8)^−0.375^ × 0.995^Age^ [×1.08 if black] (Scr ≤ 0.7) (Scys > 0.8), eGFR = 130 × (Scr/0.7)^−0.248^ × (Scys/0.8)^−0.711^ × 0.995^Age^ [×1.08 if black]
	Female (Scr > 0.7) (Scys ≤ 0.8), eGFR = 130 × (Scr/0.7)^−0.601^ × (Scys/0.8)^−0.375^ × 0.995^Age^ [×1.08 if black] (Scr > 0.7) (Scys > 0.8), eGFR = 130 × (Scr/0.7)^−0.601^ × (Scys/0.8)^−0.711^ × 0.995^Age^ [×1.08 if black]
	Male (Scr ≤ 0.9) (Scys ≤ 0.8), eGFR = 135 × (Scr/0.9)^−0.207^ × (Scys/0.8)^−0.375^ × 0.995^Age^ [×1.08 if black] (Scr ≤ 0.9) (Scys > 0.8), eGFR = 135 × (Scr/0.9)^−0.207^ × (Scys/0.8)^−0.711^ × 0.995^Age^ [×1.08 if black]
	Male (Scr > 0.9) (Scys ≤ 0.8), eGFR = 135 × (Scr/0.9)^−0.601^ × (Scys/0.8)^−0.375^ × 0.995^Age^ [×1.08 if black] (Scr > 0.9) (Scys > 0.8), eGFR = 135 × (Scr/0.9)^−0.601^ × (Scys/0.8)^−0.711^ × 0.995^Age^ [×1.08 if black]

BIS1 [81]	3736 × creatinine^−0.87^ × age^−0.95^ × 0.82 (if female)

BIS2 [81]	767 × cystatin C^−0.61^ × creatinine^−0.40^ × age^−0.57^ × 0.87 (if female)

eCCr: estimated creatinine clearance; Scr: serum creatinine; BUN: blood urea nitrogen; Scys: serum cystatin C; MDRD: Modification of Diet in Renal Disease; CKD-EPI: Chronic Kidney Disease Epidemiological Collaboration; BIS: Berlin Initiative Study.
